# Haematopoietic ESL-1 enables stem cell proliferation in the bone marrow by limiting TGFβ availability

**DOI:** 10.1038/ncomms10222

**Published:** 2016-01-08

**Authors:** Magdalena Leiva, Juan A. Quintana, José M. Ligos, Andrés Hidalgo

**Affiliations:** 1 Area of Developmental and Cell Biology, Centro Nacional de Investigaciones Cardiovasculares, 28029 Madrid, Spain; 2 Cellomics Unit, Centro Nacional de Investigaciones Cardiovasculares, 28029 Madrid, Spain; 3 Institute for Cardiovascular Prevention, Ludwig-Maximilians University, 80336 Munich, Germany

## Abstract

The life-long maintenance of haematopoietic stem and progenitor cells (HSPCs) critically relies on environmental signals produced by cells that constitute the haematopoietic niche. Here we report a cell-intrinsic mechanism whereby haematopoietic cells limit proliferation within the bone marrow, and show that this pathway is repressed by E-selectin ligand 1 (ESL-1). Mice deficient in ESL-1 display aberrant HSPC quiescence, expansion of the immature pool and reduction in niche size. Remarkably, the traits were transplantable and dominant when mutant and wild-type precursors coexisted in the same environment, but were independent of E-selectin, the vascular receptor for ESL-1. Instead, quiescence is generated by unrestrained production of the cytokine TGFβ by mutant HSPC, and *in vivo* or *in vitro* blockade of the cytokine completely restores the homeostatic properties of the haematopoietic niche. These findings reveal that haematopoietic cells, including the more primitive compartment, can actively shape their own environment.

## Abstract (web summary)

Hematopoietic stem and progenitor cell (HSPCs) proliferation is controlled by signals from the niche. Here, Leiva *et al*. show *in vivo* in mice that deletion of E-selectin ligand 1 causes quiescence of HSPCs and a reduction in niche size, which is mediated by changes of TGFß levels in the bone marrow.

## Graphical abstract

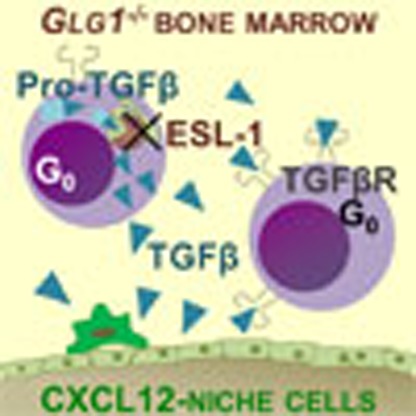

Quiescence, an essential feature of haematopoietic stem cells (HSCs), is thought to prevent exhaustion of the most primitive compartment and to ensure protection from environmental stress and DNA-damaging agents^1^. Imaging and computational analyses have revealed that mesenchymal perivascular cells around bone marrow (BM) arterioles promote cycle arrest on HSC^2^. These arteriolar niches are in turn innervated by nerves ensheathed by Schawnn cells, which also contribute to cycle arrest and preservation of HSC *in vivo*^3^. To preserve a homeostatic balance, however, the environment must also provide HSC with proliferative signals. Among niche cells, endothelial cells promote HSC proliferation through E-selectin^4^. Whether proliferative regulation can also originate from non-stromal cells, including haematopoietic cells that are an integral part of the BM niche, remains an outstanding question.

HSC, which by definition are located within the niche, can produce cytokines that impact their own proliferation and differentiation^5^. Among these cytokines, transforming growth factor β (TGFβ) has been implicated in the regulation of haematopoietic stem and progenitor cell (HSPC) cycling by inhibiting lipid raft clustering and cytokine signalling^6^. The impact of this cytokine for the *in vivo* maintenance of HSC is highlighted by the loss of both quiescence and function of HSC lacking the TGFβ receptor II, or by analysis of animals in which TGFβ-producing Schwann cells were eliminated by sympathetic denervation^3^. Defining the mechanisms that regulate TGFβ production is therefore essential to understand how maintenance of HSPC in ensured *in vivo*.

In this study, while searching for the ligand responsible for relaying the proliferative signals that emerge from vascular-borne E-selectin on HSPC, we found that the absence of E-selectin ligand 1 (ESL-1), a major ligand for E-selectin on haematopoietic cells^7^, results in global quiescence in the primitive haematopoietic compartment. Unexpectedly, we show that ESL-1 maintains homeostatic proliferation within the BM not by engaging E-selectin, but by repressing the production of TGFβ by haematopoietic cells, including HSPC.

## Results

### ESL-1-deficiency causes HSPC quiescence and expansion

E-selectin expressed by vascular cells induces homeostatic proliferation of HSC by signalling through unknown receptor(s) on haematopoietic precursors that are distinct from its canonical ligands PSGL-1 and CD44 (ref. 4). We hypothesized that ESL-1 could be endowed with the capacity of transducing the proliferative signals from the BM vasculature because it is a dominant E-selectin ligand of haematopoietic progenitors, in which it promotes homing to the BM^7^. Indeed, we could detect abundant ESL-1 transcript and protein in phenotypically immature HSPC (Supplementary Fig. 1a,b), suggesting that ESL-1 might be functionally relevant in the most primitive haematopoietic compartment.

**Figure 1:**
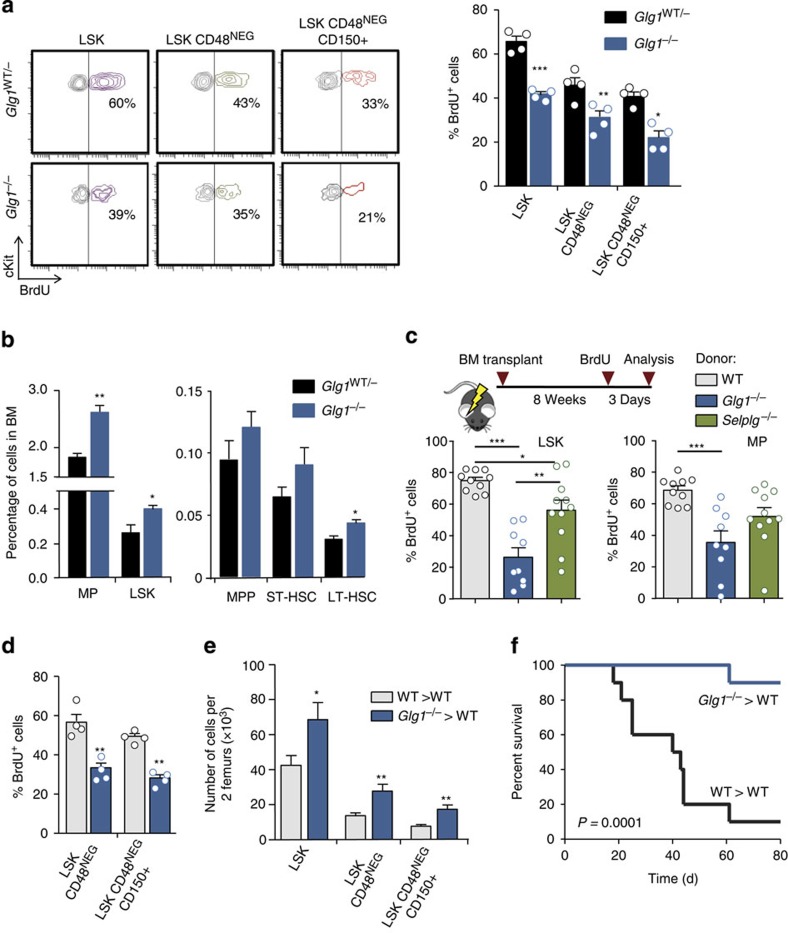
ESL-1 controls the proliferation and numbers of HSPC in the bone marrow. (**a**) Representative plots of BrdU proliferation assays gated on Lineage^NEG^ cKit^HI^ Sca1^+^ (LSK) cells, LSK CD48^NEG^ and LSK CD48^NEG^ CD150^+^ cells in *Glg1*^−/−^ and *Glg1*^WT/−^ littermates. Right, percentage of BrdU-incorporating cells; *n*=4. (**b**) Percentage of the indicated populations in the BM of *Glg1*^−/−^ and *Glg1*^WT/−^ littermates; *n*=5. (**c**) Experimental design and percentage of BrdU incorporation in WT, *Glg1*^−/−^and *Selplg1*^−/−^ progenitors in transplanted WT mice; *n*=9–11. (**d**) Percentage of BrdU-incorporating cells in the indicated HSPC populations from WT and *Glg1*^−/−^-transplanted mice; *n*=4. (**e**) Absolute numbers of the indicated populations in two femurs of WT and *Glg1*^−/−^ transplanted mice; *n*=7. (**f**) Survival curves of mice transplanted with WT or *Glg1*^−/−^ BM cells after repeated injection of 5-FU; *n*=10. *P<0.05; **P<0.01; ***P<0.005 as determined by Student's *t*-test for two data sets, or one-way analysis of variance with Turkey's test for multiple data sets. In **f**, comparison of survivals was performed using the Log-rank test. Each circle represents a mouse. Bars show mean±s.e.m.

To test the possibility that ESL-1 promoted HSPC proliferation, we measured cycling of haematopoietic precursors in mice deficient in ESl-1 (*Glg1*^−/−^)^8^ and heterozygous littermates using 5-bromodeoxyuridine (BrdU) incorporation assays. *Glg1*^−/−^ mutants displayed reductions in the frequencies of proliferating Lineage^NEG^ cKit^HI^ Sca1^+^ (LSK) cells and the more primitive LSK CD48^NEG^ and LSK CD48^NEG^CD150^+^ precursors (Fig. 1a). We obtained independent confirmation of a reduced frequency of cycling *Glg1*^−/−^ LSK CD48^NEG^CD150^+^ precursors by staining for Ki67, a nuclear marker of cell cycling (Supplementary Fig. 1c). In parallel to cycle arrest, we found an increase in the frequency of myeloid progenitors (Lineage^NEG^ cKit^HI^ Sca1^NEG^; MPs) and LSK cells, as well as in primitive LSK CD135^NEG^CD90^+^ cells (LT-HSC) in these mutants (Fig. 1b). Contrasting with the expansion of immature precursors, we did not find increased numbers of mature myeloid cells in the BM of ESL-1-deficient mice, with the exception of resident macrophages which were expanded (Supplementary Fig. 2a). Blood leukocyte counts were slightly elevated but normal for erythrocyte and platelet numbers, and the mice presented a mild splenomegaly (Supplementary Fig. 2b). Collectively, these data indicated that ESL-1 controls HSPC cycling and numbers, suggesting that ESL-1 might be the E-selectin ligand responsible for transducing proliferative signals *in vivo*.

### Quiescence of *Glg1* ^−/−^ HSPC is transplantable and dominant

We reasoned that if ESL-1 triggered HSPC proliferation by engaging E-selectin, then reconstitution of *Glg1*^−/−^ haematopoiesis into WT recipient mice should recapitulate the phenotype of the full mutants. If this was the case, these haematopoietic chimeras would also allow a more thorough examination of mutant HSPC given the extremely low birth rate of *Glg1*^−/−^ mice (<2% of the 25% expected from heterozygous breeders)^7^. WT mice reconstituted with *Glg1*^−/−^ donor cells recovered well, with blood counts and splenomegaly similar to full mutants, and BM cellularity similar to mice transplanted with WT donors (Supplementary Fig. 2c). As predicted, mice reconstituted with mutant cells reproduced the marked reductions in proliferating LSK and MP cells (Fig. 1c), as well as primitive LSK CD48^NEG^ and LSK CD48^NEG^CD150^+^ cells (Fig. 1d); in contrast, mice deficient in a different E-selectin ligand, PSGL-1 (*Selplg*^−/−^), only displayed mild reductions in proliferation that were restricted to LSK cells (Fig. 1c). Importantly, mice reconstituted from *Glg1*^−/−^ donors also displayed increased quiescence as shown by reductions in Ki67 staining in LSK cells (Supplementary Fig. 2d) and increased number of surviving reconstituting HSPC after hydroxyurea treatment (∼4-fold increase) that was markedly higher than that seen in untreated mice (Supplementary Fig. 2e). Transplanted mice reproduced the expansion in *Glg1*^−/−^ mice of primitive progenitors (Fig. 1e), including a ∼2.5-fold increase in the number of functional HSC as assessed by long-term reconstitution using limiting dilution assays (Supplementary Fig. 2f). The dramatic increase in *Glg1*^−/−^ repopulating units in the hydroxyurea suicide assay over the wild-type (WT) group confirmed elevated quiescence of mutant HSPC even if the starting number of progenitors was higher in mice reconstituted with *Glg1*^−/−^ BM cells. To test the functional consequence of quiescence in *Glg1*^−/−^ progenitors, we induced haematopoietic exhaustion by repeated administration of the chemotherapeutic agent 5-fluorouracil (5-FU). Although the majority of mice reconstituted from WT donors succumbed by 8 weeks, mice transplanted with *Glg1*^−/−^ marrow showed a dramatic resistance to exhaustion and death (Fig. 1f). These results provide functional support to the observation that ESL-1 promotes homeostatic proliferation of HSPC.

**Figure 2:**
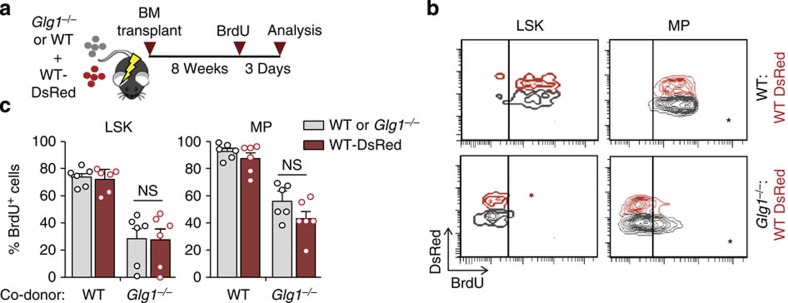
Deficiency in haematopoietic ESL-1 generates a quiescent bone marrow environment. (**a**) Experimental scheme for the generation of mixed chimeric mice by bone marrow transplantation. (**b**) Representative plots of BrdU incorporation in LSK and myeloid progenitors (MPs) in WT:WT-DsRed or *Glg1*^−/−^:WT DsRed chimeric mice. (**c**) Percentage of BrdU-incorporating WT-DsRed and *Glg1*^−/−^ cells in the mixed chimeric mice shown in **b**; *n*=6. NS, not significant. Each circle represents a mouse. Bar graphs show mean±s.e.m.

To delineate whether the phenotype was cell-autonomous as predicted by our model, we co-transplanted WT (expressing the DsRed reporter gene) together with *Glg1*^−/−^ BM cells into WT recipients, and after 8 weeks analysed HSPC proliferation (Fig. 2a). Unexpectedly, not only ESL-1-deficient progenitors displayed the expected reductions in proliferation, but WT progenitors sharing the same host as *Glg1*^−/−^ mutants displayed identical reductions, which were not seen when the co-transplanted cells were of WT origin (Fig. 2b,c). These observations indicated that the proliferative phenotype of ESL-1-deficient haematopoietic precursors was transplantable, and that ESL-1 cells transferred their proliferative trait to genetically normal HSPC that share the same microenvironment.

### Elevated TGFβ triggers quiescence in the absence of ESL-1

**Figure 3:**
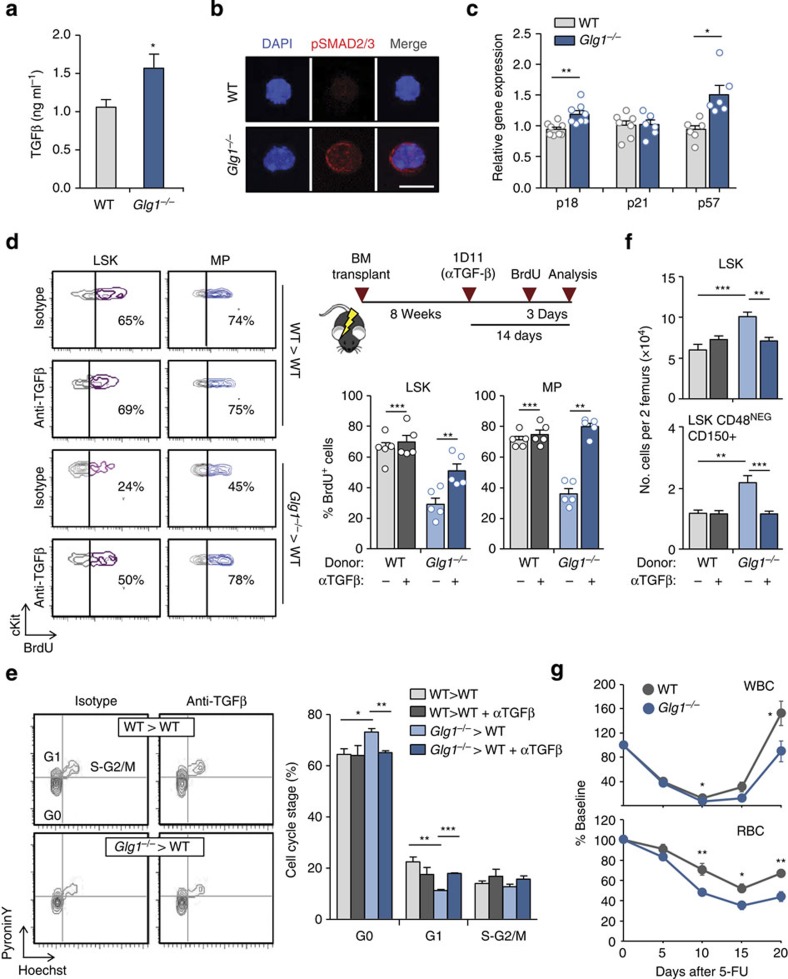
ESL-1 controls HSPC proliferation by repressing TGFβ secretion. (**a**) TGFβ levels in the BM of WT and *Glg1*^−/−^-transplanted mice; *n*=8. (**b**) Micrographs of purified WT and *Glg1*^−/−^ LSK cells stained for pSMAD2/3 (red) and DAPI (blue). Scale bar, 5 μm. Data are from two independent experiments. (**c**) Expression of p18, p21 and p57 in sort-purified WT and *Glg1*^−/−^ LSK cells; *n*=6–8. (**d**) Representative contour plots of BrdU incorporation in WT and *Glg1*^−/−^ LSK cells and MP treated with anti-TGFβ or control antibody. Right, experimental design (top) and bar graphs (bottom) show the percentage of BrdU^+^ cells in the different groups; *n*=5. Each circle represents a mouse. (**e**) Representative plots and quantification of cell cycle analyses of WT and *Glg1*^−/−^ LSK cells and MP from mice treated with anti-TGFβ or control antibody; *n*=6. (**f**) Absolute numbers of the indicated progenitor populations in mice reconstituted with WT or *Glg1*^−/−^ marrow, and treated or not with anti-TGFβ antibody; *n*=5. (**g**) Recovery kinetics of leukocytes (WBC) and erythrocytes (RBC) in the blood of mice reconstituted with WT or *Glg1*^−/−^ BM after treatment with a single dose of 5-FU; *n*=10. *P<0.05; **P<0.01; ***P<0.001 determined by Student's test (**a**,**g**), Wilcoxon matched-pairs (**c**) or one-way analysis of variance with Turkey's test (**d**,**e**,**f**). Data are show as mean±s.e.m.

The dominant phenotype of *Glg1*^−/−^ mutants indicated that E-selectin-derived signals could not fully account for the proliferative arrest. We thus searched for alternative mechanisms for this unexpected phenotype. ESL-1 has been shown to limit the maturation and secretion in chondrocytes of TGBβ^8^, a cytokine with potent anti-proliferative effects on HSC^9^,^10^. We thus tested whether abnormal TGFβ signalling might account for the proliferative arrest observed in the absence of ESL-1. In agreement with this possibility, TGFβ protein levels were elevated in mice reconstituted with *Glg1*^−/−^ marrow, as well as in full *Glg1*^−/−^ mutants (Fig. 3a and Supplementary Fig. 3a), and these elevations correlated with enhanced phosphorylation of Smad2/3 in LSK cells (Fig. 3b and Supplementary Fig. 3b). In addition, transcriptomic analyses of WT and mutant HSPC revealed a pattern of expression of cycle inhibitory genes (Fig. 3c) that was fully consistent with the temporal activation of the TGFβ signalling pathway in the context of BM regeneration^11^. To determine whether elevated TGFβ signalling was responsible for HSPC quiescence in the absence of ESL-1, we treated mice transplanted with mutant or WT BM with an anti-TGFβ antibody that efficiently blocks the cytokine *in vivo*^12^. Inhibition of TGFβ for 2 weeks resulted in restoration of HSPC proliferation in *Glg1*^−/−^ mutants as measured by BrdU incorporation and cell cycle analyses (Fig. 3d,e). Importantly, TGFβ inhibition also restored progenitor numbers (Fig. 3f) without affecting BM cellularity or other haematological parameters, or the proliferative status of HSPC in WT cells (Fig. 3d,e).

To test whether increased TGFβ signalling in these mutants could influence recovery under haematopoietic stress, we next assessed the kinetics of haematopoietic regeneration after 5-FU treatment in mice bearing WT or *Glg1*^−/−^ BM. We found a delay in the recovery of circulating leukocytes and erythrocytes in the absence of ESL-1 (unpaired *t*-test; Fig. 3g), and a similar trend for platelet recovery (Supplementary Fig. 3c), which were in agreement with the reduced proliferative rate in mutant BM. The delayed recovery in *Glg1*^−/−^ mutants contrasted with their enhanced resistance to repeated 5-FU treatment (Fig. 1f), which was fully consistent with the notion that quiescence protects from haematopoietic exhaustion upon chronic stress^4^. Interestingly, the recovery delays became noticeable after day 10, a time when elevated TGFβ levels are known to return to baseline after 5-FU treatment^11^, and blockade of TGFβ normalized the recovery kinetics of all blood parameters in the *Glg1*^−/−^ group (Supplementary Fig. 3c). Thus, regulation of TGFβ levels in the BM is orchestrated by ESL-1, and this control preserves homeostatic proliferation of HSPC and facilitates haematopoietic recovery upon stress.

### ESL-1 controls proliferation independently of E-selectin

**Figure 4:**
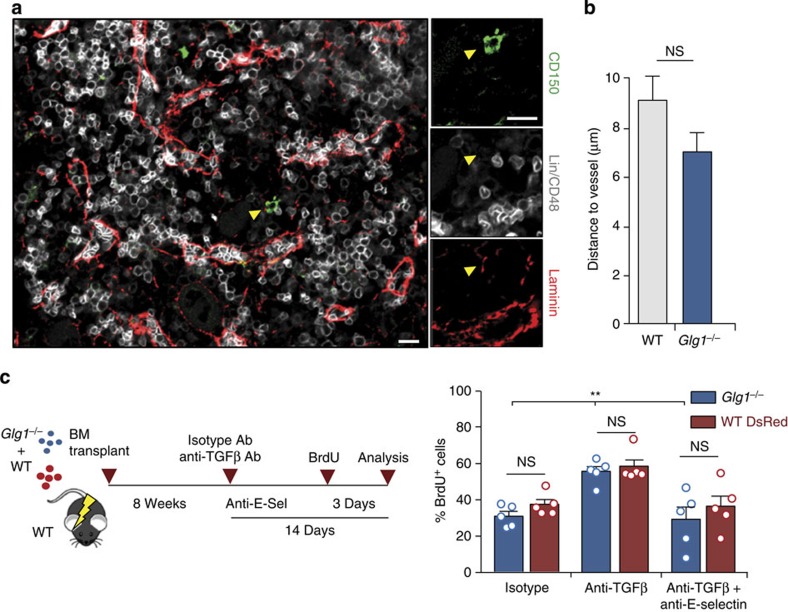
ESL-1 and E-selectin induce HSPC proliferation through independent mechanisms. (**a**) Representative images of the distribution of Lin^NEG^ CD48^NEG^ CD150^+^ cells in the BM relative to laminin^HI^ (red) vessels; arrowheads indicate the position of a scored cell. Scale bars, 10 μm. *n*=7–9 mice, 55–70 cells per group. (**b**) Distances of Lin^NEG^ CD48^NEG^ CD150^+^ cells to vessels in WT or *Glg1*^−/−^ BM. NS, not significant, as determined by Student's *t*-test. *n*=39 or 40 cell per group. (**c**) Experimental design and percentage of BrdU^+^ LSK cells from each donor (WT-DsRed or *Glg1*^−/−^) in mixed chimeric mice treated with isotype-matched, anti-TGFβ alone or anti-TGFβ plus anti-E-selectin antibodies. *n*=5. Bars show mean±s.e.m. Each circle represents a mouse. **P<0.01 by one-way analysis of variance with Turkey's test comparing *Glg1*^−/−^cells across the different groups; NS, not significant.

Our findings did not rule out a possible coordination between vascular E-selectin and ESL-1 in inducing proliferation. We therefore asked whether, in addition to regulating TGFβ levels, ESL-1 controlled the distribution of HSPC through binding E-selectin on sinusoidal vessels, as quiescence is favoured in distinct perivascular regions of the marrow^2^. To address this possibility, we examined the distribution of Lin^NEG^ CD48^NEG^ CD150^+^ cells or Lin^NEG^ cKit^+^ progenitors in the BM of mice reconstituted with WT or *Glg1*^−/−^ donors (Fig. 4a,b and Supplementary Fig. 4), and scored the distance between each progenitor and the nearest vascular structure. We found that WT and *Glg1*^−/−^ progenitors displayed identical distribution within the marrow (Fig. 4a,b and Supplementary Fig. 4), indicating that alterations in progenitor distribution were unlikely to account for the proliferative defects of mutant mice.

To further examine whether E-selectin and ESL-1 functioned along the same pathway to prevent quiescence, we generated mixed chimeric mice using WT-DsRed^+^ and *Glg1*^−/−^ donors that were co-transplanted into WT mice (Fig. 4c). Using this approach, we generated two coexisting populations of HSPC displaying similar proliferative defects (Fig. 2), in which we tested the capacity of TGFβ blockade to restore proliferation. If E-selectin signalled through ESL-1, we predicted that this treatment should rescue proliferation only in WT HSPC. TGFβ blockade, however, restored proliferation of both populations of HSPC (Fig. 4c). In addition, blockade of TGFβ in E-selectin-deficient recipients (Supplementary Fig. 5) or blockade of both E-selectin and TGFβ in WT recipients (Fig. 4c) failed to restore proliferation of WT or *Glg1*^−/−^ HSPC. These experiments demonstrated that E-selectin and ESL-1 control proliferation through different mechanisms.

### Haematopoietic precursors are a relevant source of TGFβ

**Figure 5:**
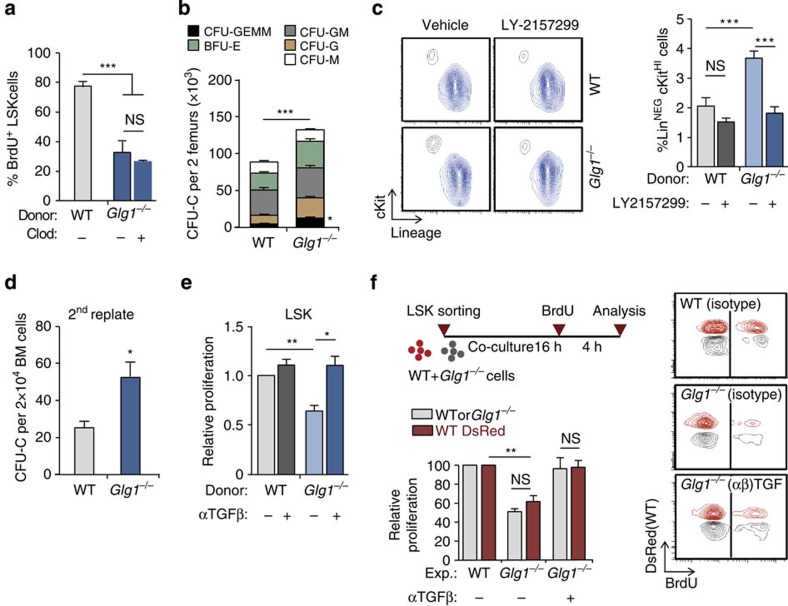
Haematopoietic precursors are a relevant source of TGFβ. (**a**) Percentage of BrdU incorporation in WT or *Glg1*^−/−^ LSK cells of control or macrophage-depleted (Clod) mice; *n*=5. (**b**) Frequency of the different types of CFU-C in mice reconstituted with WT or *Glg1*^−/−^ donors; *n*=6. (**c**) Contour plots (left) and frequency (right) of Lin^NEG^ cKit^+^ cells obtained from WT or *Glg1*^−/−^ marrow grown in the presence or absence of the TGFβRI inhibitor LY-2157299; *n*=6. (**d**) Number of secondary colonies after replating WT or *Glg1*^−/−^ CFU-C from primary cultures; *n*=3 independent experiments. (**e**) Relative *in vitro* proliferation of WT or *Glg1*^−/−^ LSK cells, as measured by BrdU incorporation; *n*=3 independent experiments. (**f**) Experimental design to test the *in vitro* proliferation of WT and *Glg1*^−/−^ LSK cells in co-cultures with or without TGFβ blockade. Representative contour plots (right) and quantification of the relative proliferation; *n*=3 independent experiments. Data are shown as mean±s.e.m.; *P<0.05; **P<0.01; ***P<0.001; NS, not significant, as determined by Student's *t*-test (**b**,**d**), or one-way analysis of variance with Turkey's test (**a**,**c**,**e** and **f**).

The transplantation experiments suggested that the elevations in the levels of TGFβ in the *Glg1*^−/−^ group originated from the haematopoietic compartment. To search for the relevant source of TGFβ among haematopoietic cells, we measured ESL-1 protein in multiple BM populations by western blot. Myeloid leukocytes and immature progenitors displayed the highest levels of ESL-1 (Supplementary Fig. 6a). Because BM-resident macrophages were expanded in *Glg1*^−/−^ mice (Supplementary Fig. 2a) and are a potential source of TGFβ^13^, we depleted these cells using clodronate liposomes and measured the impact on HSPC proliferation. Despite efficient depletion of macrophages (Supplementary Fig. 6b), this treatment failed to restore HSPC proliferation of *Glg1*^−/−^ HSPC (Fig. 5a), thus excluding these cells as a relevant source of TGFβ.

We then focused on immature haematopoietic precursors, which also expressed high levels of intracellular ESL-1 (Supplementary Fig. 7a) and produce TGFβ^5^. Given the lack of tools for specific genetic or pharmacological manipulation of HSPC *in vivo*, we performed *ex vivo* analyses. We first noticed that *Glg1*^−/−^ marrow cells grown in semisolid cultures yielded more CFU-C (Fig. 5b) that contained, however, less cells than WT colonies (Supplementary Fig. 7b). Treatment with the TGFβR-I inhibitor LY-2157299 reverted the cellularity of *Glg1*^−/−^ colonies without affecting WT-derived CFU-C (Supplementary Fig. 7b). *Glg1*^−/−^ cultures also yielded an increase in the frequency of Lin^NEG^ cKit^HI^ progenitors after 7 days of culture, which could also be reverted by the TGFβRI inhibitor (one-way analysis of variance with Tukey's multigroup test; Fig. 5c), and replating assays resulted in elevated numbers of secondary colonies, suggesting a higher self-renewal ability of *Glg1*^−/−^ progenitors (Fig. 5d). We could reproduce these results with sort-purified LSK cells, whose proliferative capacity was reduced in *Glg1*^−/−^ cells and this could also be reverted by blocking TGFβ (Fig. 5e and Supplementary Fig. 7c), indicating that early progenitors are an active source of inhibitory TGFβ. Importantly, and agreeing with the role of ESL-1 in limiting posttranslational processing of pro-TGFβ^8^, we did not find changes in *Tgfb1* transcript levels in mutant LSK cells (Supplementary Fig. 8a), and in contrast found mild elevations in the levels of latent TGFβ on the surface of *Glg1*^−/−^ progenitors compared with WT cells (Supplementary Fig. 8b).

Because *Glg1*^−/−^ cells induced quiescence on neighbouring WT HSPC *in vivo* (Fig. 2), we sought to reproduce this dominance *in vitro* using purified LSK cells. Mixed cultures of WT and *Glg1*^−/−^ LSK cells resulted in reduced proliferation of both types of progenitors, compared with mixtures of WT cells only. Importantly, blockade of TGFβ reversed the proliferative defects of both WT and *Glg1*^−/−^ cells (Fig. 5f). Altogether, these observations indicated that HSPC are a relevant source of TGFβ, which acts on neighbouring progenitors to block proliferation.

### Haematopoietic ESL-1 maintains CXCL12-producing niche cells

**Figure 6:**
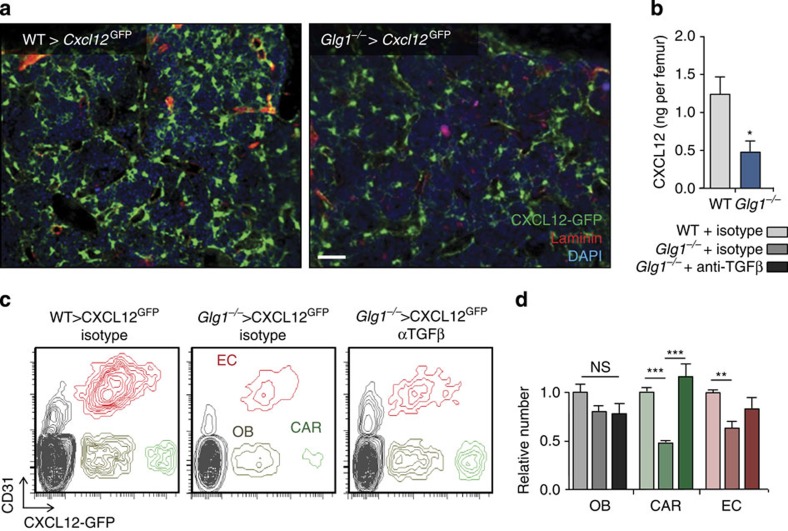
Haematopoietic ESL-1 preserves the CXCL12-producing bone marrow stroma. (**a**) Whole-mount staining of sternal BM of *Cxcl12*^GFP^ mice transplanted with WT or *Glg1*^−/−^ bone marrow cells; *n*=4. Scale bar, 50 μm. (**b**) CXCL12 protein levels in the BM mice transplanted with WT or *Glg1*^−/−^ bone marrow cells; *n*=5. (**c**) Representative density plots showing osteoblasts (OB), CAR and endothelial cells (ECs) in the bone marrow of the BM of *Cxcl12*^GFP^ mice reconstituted with WT or *Glg1*^−/−^ BM, and treated or not with an anti-TGFβ antibody for 2 weeks, and quantification of their relative numbers (**d**); *n*=5 mice per group. Data are shown as mean±s.e.m.; *P<0.05; **P<0.01; ***P<0.001 as determined by Student's *t*-test (**b**), or one-way analysis of variance with Turkey's test (**d**).

We finally explored whether the repressive signals originating from *Glg1*^−/−^ haematopoietic cells could affect the stromal niche. To this end, we transplanted WT or *Glg1*^−/−^ donors into *Cxcl12*^GFP^ reporter mice, which allow identification of perivascular cells expressing high levels of the chemokine (CD45^NEG^ CD31^NEG^ GFP^HI^; CAR cells), as well as endothelial cells (CD45^NEG^ CD31+ GFP^LO^) and osteoblasts (CD45^NEG^ CD31^NEG^ GFP^LO^)^14^,^15^. Imaging of sternal preparations revealed reductions in CXCL12-producing cells in the *Glg1*^−/−^ group (Fig. 6a), and this correlated with strong reductions in CXCL12 protein in the BM (Fig. 6b). Cytometric analyses revealed that these reductions were associated with marked decreases in the number of CAR and endothelial cells, but not osteoblasts (Fig. 6c,d). Importantly, the reductions in CAR and endothelial cells could be corrected by blocking TGFβ (Fig. 6d), and correlated with elevated TGFβ-induced signalling in these two cell types but not on osteoblasts (Supplementary Fig. 9a,b), suggesting that this cytokine produced by ESL-1-deficient haematopoietic cells can directly suppress the stromal niche, as supported by previous reports^16^. Altogether, the data suggested that haematopoietic-borne ESL-1 can control HSPC proliferation directly through cytokine secretion, and/or indirectly through repressive effects on supportive niche cells.

## Discussion

Although the list of stromal constituents of the niche is being rapidly refined^17^, only a handful of studies have focused on haematopoietic cells as functional niche elements that regulate the fate of the HSC from which they originate. In this study, we show that haematopoietic progenitors have the capacity to regulate the proliferative status of neighbouring HSPC through production of TGFβ and that this function is limited by ESL-1. Our findings reveal that regulation of the niche can originate from haematopoietic cells, including HSPC, and suggest that the partnership of haematopoietic and mesenchymal stem cells that are at the core of the BM niche^18^ allows mutual regulation of both partners, as has been evidenced in leukaemic models^19^,^20^.

Besides HSPC, other haematopoietic cells including macrophages^21^,^22^ and megakaryocytes^23^,^24^ may be relevant sources of TGFβ and may thus regulate niche size and HSPC proliferation. Although we have excluded macrophages as a major source, perivascular megakaryocytes, which produce abundant TGFβ^24^, could also have a strong impact on the global levels of the cytokine in the BM in the absence of ESL-1. Nonetheless, we provide evidence that HSPC, which were shown in early *in vitro* studies to be an autocrine source of TGFβ^25^, can function as regulators of their own environment. This finding is particularly relevant because these cells are by definition the only population unambiguously located within a haematopoietic niche.

An important extension from our study will be to uncover the physiological or pathological scenarios in which the regulatory restraint imposed by ESL-1 becomes inactive. As under steady-state conditions blockade of the TGFβ pathway does not alter HSC proliferation (this study and^11^), we propose two possible scenarios in which loss of this regulation may be relevant: ageing and stress. The finding that *Glg1*^−/−^ mice display a myeloid bias and display delayed recovery from stress parallels changes described in the aged haematopoietic system^26^. Interestingly, alterations in TGFβ signalling have been associated with HSC ageing and are likely to underlie the myeloid expansion in old animals^27^,^28^. An additional scenario, pathological stress, is also of interest in light of recent work showing that a transient wave of TGFβ in the stressed marrow prompts the return of HSC to quiescence after an initial proliferative phase^11^, suggesting that pathways that regulate TGFβ signalling may be important for restoring haematopoietic homeostasis. Both scenarios are currently being tested in our laboratory.

The dominant effect that ESL-1 exerts on neighbouring HSPC has implications for understanding the interactions of stem cells within their niche. It suggests that deregulation of the pathway in a single cell could initiate alterations in restricted populations of HSPC and stromal cells that share a common ‘microniche'. Whether clustering represents the normal distribution of HSPC *in vivo* is unclear, but the recent identification of hemospheres as units of clonal expansion^29^ supports this possibility. Also noteworthy is the finding that subsets of stromal niche cells associated with myeloid or the most primitive precursors (endothelial and CAR cells^17^,^30^) appear repressed in the absence of ESL-1, whereas osteoblasts that are associated with the lymphoid lineage that expresses little ESL-1 remain largely unaffected, suggesting local regulation of the various haematopoietic environments.

An unexpected finding from our study was that, although ESL-1 has been shown to be a ligand for E-selectin on haematopoietic progenitors^7^, each molecule (ESL-1 and E-selectin) affects HSPC proliferation through independent mechanisms. The predominant expression of ESL-1 inside the cell rather than at the surface (which would be required for selectin binding) is consistent with this independent mechanism. Thus, the identity of the relevant E-selectin ligand(s) on HSPC responsible for the proliferative effects remains unknown, although it is possible that glycosphingolipids, or a combination of various glycoproteins (as shown for the recruitment of neutrophils^31^), cooperate for selectin binding and for cycle arrest. This possibility is sustained by the growing appreciation that a complex array of differentially glycosylated proteins (and lipids) other than PSGL-1 and ESL-1 can function as ligands for E-selectins on haematopoietic cells^7^,^32^. This important issue deserves further study. In addition, although it has been speculated that E-selectin might control HSPC by dictating their distribution within the non-uniform BM microenvironment^4^, the mechanism by which this selectin and its ligand(s) ultimately regulate HSPC proliferation remains to be elucidated.

In summary, the identification of an intrinsic pathway controlled by ESL-1 that regulates HSPC proliferation, but can also impact the behaviour of neighbouring stromal cells and HSPC (scheme in Supplementary Fig. 10), yields important insights into how stem cell dynamics are regulated to maintain homeostasis within the BM.

## Methods

### Mice

All experiments were performed in 6- to 10-week-old male mice housed in a specific pathogen-free facility. ESL-1- (*Glg1*^−/−^), PSGL-1- (*Selplg*^−/−^) and E-selectin- (*Sele*^−/−^) deficient mice were obtained from B. Lee (Baylor College of Medicine, Houston, TX, USA) and P. Frenette (Einstein College of Medicine, NY, USA), and have already been described^8^,^33^. *Cxcl12-Gfp* knock-in mice^34^ were also used as recipients. Mice expressing *DsRed* under the β-actin promoter were used as donors in some experiments. Wild-type C57BL/6 (CD45.2^+^) and congenic B6.SJL (CD45.1^+^) mice were used as donors or recipients. All mice were in a pure C57BL/6 background. Experimental procedures were approved by the Animal Care and Ethics Committee at Fundación CNIC and Comunidad de Madrid.

### Cell isolation

After euthanasia, femurs were collected and flushed into ice-cold PBS plus 0.5% fetal bovine serum (FBS) for the isolation of BM cells. For estimation of CXCL12-expressing niche cells, femurs were flushed and incubated with 1 U ml^−1^ liberase (Roche Applied Science) and 12 mU ml^−1^ DNase I (Sigma) in HBSS for 30 min at 37 °C. Cells were sorted on a FACSAria (BD Biosciences) to >95% purity.

### Flow cytometry

For BM mature myeloid compartment, cells were stained with fluorescein isothiocyanate (FITC)-conjugated Gr-1 (Clone RB6-8C5), PE-conjugated CD115 and Alexa Fluor 647-conjugated F4/80 (Clone CI:A3-1; purchased from Serotec) antibodies in PBS containing 2 mM EDTA and 0.5% FBS. For LSKs and MPs, cells were stained with biotinylated lineage antibody cocktail (CD3ɛ, B220, CD11b, Gr1 and Ter119) from BD Bioscience, together with streptavidin conjugated to DyLight 405 (purchased from Jackson), antibody to Sca-1 (Clone: D7) conjugated to FITC or antigen-presenting cell (APC) and antibody to c-Kit (Clone: 2B8) conjugated to PE-Cy7. For the analyses of MPP, ST-HSC and LT-HSC cells, samples were stained with the biotinylated lineage antibody cocktail plus anti-Sca-1-FITC, anti-cKit-PE-Cy7, anti-CD135-PE (Clone: A2F10) and anti-CD90.2-APC (Clone: 53-2.1). For HSC staining using the SLAM markers, cells were stained with biotinylated lineage antibody cocktail and biotinylated CD48 (Clone: HM48-1) together with streptavidin conjugated to Alexa Fluor 405, and antibodies against Sca-1-APC, c-Kit-PE-Cy7 and CD150-PE (Clone: TC15-12F12.2). CD48 and CD150 antibodies were purchased from BioLegend. All other antibodies were purchased from eBioscience. For assessment of multilineage chimerism, blood leukocytes were stained with PE-Cy7-conjugated CD45.1 (Clone A20), purchased from Southern Biotech, FITC-conjugated CD45.2 (Clone 104; TOMBO Bioscience), PE-conjugated CD11b (Clone M1/70; eBioscience), APC-conjugated CD3ɛ (Clone 145-2C11; BioLegend) and biotinylated-B220 together with streptavidin conjugated to DyLight 405.

For BrdU staining of HSC, BM cells were first enriched by immunomagnetic depletion using BD IMag kit (BD Bioscience). Lineage negative enriched cells were stained with biotinylated CD48 together with streptavidin conjugated to Alexa Fluor 405, plus antibodies against Sca-1-APC, c-Kit-PE-Cy7 and CD150-PE. For BrdU staining of LSK cells and MP, cells were directly stained as above. Stained cells were then fixed and permeabilized using the Cytoperm/Cytofix kit (BD Biosciences) as per the manufacturer's instructions and incubated with DNAse I at 37 °C for 1 h. Cells were finally stained with anti-BrdU-APC before cytometric analysis.

For detection of Ki67 in LSK or SLAM progenitors, BM suspensions were surface stained as above, fixed and permeabilized using the Mouse Foxp3 fixation and permeabilization buffers (BD Biosciences), and then stained with an anti-mouse Ki67 conjugated to Alexa600 (Clone: SolA15; eBioscience) and Hoechst 33342.

To analyse cell cycle on LSK cells and MP, live cells were surface stained with the lineage antibody cocktail together with streptavidin conjugated to APC-Cy7, plus antibodies against Sca-1-FITC and c-Kit-APC. Cells were then stained with Hoechst 33342 (Invitrogen) at 37 °C for 45 min. Pyronin Y (Sigma-Aldrich) was then added at 1 μg ml^−1^, and the cells were incubated for another 15 min at 37 °C, washed and immediately analysed using a 561-nm excitation laser to prevent bleeding into all other channels used for this staining.

CXCL12-expressing niche cells were analysed by staining digested BM suspensions from *Cxcl12*^GFP^ mice with biotinylated antibodies against TER-119 and CD45 (Clone: 30-F11; eBioscience) followed by incubation with streptavidin conjugated to DyLight-405 and an anti-CD31-APC antibody (Clone: 390; eBioscience). High or intermediate levels of GFP in the CD45/TER119/CD31-negative fraction were used to discriminate CAR cells from the GFP^LO^ osteoblastic fraction.

To label latent TGFβ associated with the cell surface, LSK cells were stained with anti-LAP-APC (Clone: TW7-16B4) or isotype control conjugated to APC (MOPC-21; both from BioLegend). All cytometric analyses were performed using a LSR-Fortessa flow cytometer equipped with DIVA software (BD Biosciences). Data were analysed with the DIVA or FlowJo (TreeStar Inc.) softwares. All experiments were conducted at the CNIC-Cellomics Unit.

A list of all the antibodies used in this study and the dilutions at which they were used is in Supplementary Table 1.

### Generation of BM chimeras by transplantation

Donor BM cells were harvested from the appropriate genotype (WT DsRed or experimental WT, PSGL-1- or ESL-1-deficient mice) by flushing both tibiae and femora into PBS. Recipient WT C57BL/6 mice were lethally irradiated (6.5 Gy split doses, 3 h apart) before receiving 2 million BM cells by intravenous injection. For mixed chimeras, equal numbers of experimental and WT-Dsred BM cells (10^6^+10^6^ cells) were mixed and injected into the same recipients. Engraftment of recipient animals was assessed at least 8–10 weeks after transplantation by analysis of the percentage of KO and WT Dsred^+^ cells in the blood by flow cytometry. This method ensured the exclusion of variability owing to environmental factors and allowed comparing the behaviour of mutant and WT competitor cells within the same physiological environments.

### Competitive repopulation assays

5,000, 25,000, 50,000 and 100,000 WT or *Glg1*^−/−^ BM cells were injected together with 3 × 10^5^ BM cells from B6.SJL CD45.1^+^ mice into nine lethally irradiated CD45.1^+^ per dose. Multilineage chimerism was determined in the blood 16 weeks after transplantation, and mice were scored positive when donor contribution was >1%. The frequency of repopulating cells was calculated using the ELDA software (http://bioinf.wehi.edu.au/software/elda/)^35^. To assess the quiescence of HSC, we treated WT and *Glg1*^−/−^ mice with the S phase-specific cytotoxic agent hydroxyurea and harvested BM. The equivalent of 2% of one femur was mixed with 3 × 10^5^ BM cells from B6.SJL untreated mice and transplanted into lethally irradiates B6.SJL recipients. Lineage chimerism was measured at 16 weeks after transplant. The number of CD45.2^+^ reconstitution units (RUs) injected per recipient mouse was calculated using the formula (*D* × *C*)/(100–*D*), where *D* is the percentage of recipient blood leukocytes that are CD45.2^+^ at the 16-week test bleed, and *C* the number of competing CD45.1^+^ RUs that were co-injected (*C*=3, meaning that 3 × 10^5^ competing BM cells were injected). Reconstitution units per femur were then calculated. One RU is defined as the HSC content in 1 × 10^5^ BM cells^36^.

### *In vivo* treatments

For proliferation assays, BrdU (Sigma) was administered at 0.5 mg ml^−1^ in drinking water. For HSC exhaustion experiments, 5-FU (Sigma) was injected at 150 mg per kg body weight intraperitoneally (i.p.) once or every 10 days. For myelo-suppressive treatments, 5-FU was injected once (150 mg kg^−1^ i.p.). To assess haematological recovery, peripheral blood (50 μl) was collected into EDTA-coated capillary tubes (Sarstedt), and differential counts were measured using a Pentra 80 hematologic analyser (Horiba). To assess HSPC quiescence, hydroxyurea (Sigma) was injected twice (8 h apart) at 900 mg per kg body weight i.p. as described earlier^4^. For *in vivo* inhibition of TGFβ, a neutralizing antibody (clone 1D11) or a non-targeted control antibody (clone HRPN; both IgG1 from BioXcell) were intravenously injected at 100 μg per mouse once every 2 days for a total of 2 weeks. To block E-selectin, mice were treated with the same doses as above of an anti-E-selectin blocking antibody (clone 9A9; BioXcell) following the same schedule. To deplete macrophages, mice were intravenously injected with 100 μl of liposomes loaded with clodronate 8 days before analysis (Clodronateliposomes.com).

### Immunofluorescence

CXCL12-expressing niche cells and LSK cells sorted from WT of *Glg1*^−/−^ transplanted mice were cytospun onto glass slides, immediately fixed with 2% paraformaldehyde and permeabilized with cold methanol. After blocking, cells were stained with anti-pSmad2/3 (Clone: D27-F4; Cell Signalling Technology) or anti-ESL-1rabbit serum, and secondary antibody (goat anti-rabbit Alexa Fluor 555 or Alexa Fluor 488, respectively; Invitrogen). Stained cells were counterstained with 4,6-diamidino-2-phenylindole (DAPI) to reveal nuclei. LSK cells images were collected using a Leica SP5 multi-line inverted confocal microscope. CXCL12-expressing cells images were acquired with a Zeiss LSM 700 confocal system (Carl Zeiss MicroImaging).

### BM imaging

Sterna from necropsies were fixed with 4% paraformaldehyde in PBS at 4 °C overnight. Sternal bones were decalcified for 2 weeks with 0.25 M EDTA (fresh solution each 3 days) at 4 °C with agitation. To avoid the formation of crystals during the freezing, sterna were incubated with 30% sucrose in PBS for 2 days at 4 °C. The specimens were fast frozen in OCT with ethanol/dry ice and transected to obtain 2- to 3-mm fragments in a cryostat to expose the BM. BM tissues were blocked overnight to prevent nonspecific binding and permeabilized with TNB 0.1% Triton at 4 °C with horizontal shacking. BM tissues were then blocked 20 min to prevent endogenous biotin-sterptavidin conjugation (streptavidin/biotin blocking kit, Vector Labs). After three washes with PBS, we incubated with gently shaking at 4 °C overnight with goat polyclonal anti-mouse c-Kit antibody (R&D, AF1356, 1:100 dilution) or anti-mouse Collagen IV (Millipore, AB756P, 1:200 dilution), plus the lineage biotynilated cocktail containing anti-mouse Ly-6G/C-biotin (Clone RB6-8C6), anti-mouse TER-119/Ly-76-biotin, anti-mouse CD45R (B220) biotin, anti-mouse CD3e-biotin, anti-mouse CD11b/Mac-1-biotin diluted (all diluted 1:100 in PBS). After washing for 8 h under shaking with PBS plus 0.05% Triton (buffer changed each 2 h), the samples were incubated with secondary antibodies (donkey anti-goat IgG-Cy3 at 1:200 dilution, streptavidin Dylight 649 at 1:500 dilution and donkey anti-rabbit IgG-AF488 at 1:200 dilution) at 4 °C overnight with gentle shaking. Samples were then washed for 6 h with shaking as before with fresh buffer changed every 45 min, plus one wash at 4 °C with PBS for 10 min. Nuclei were stained using PBS-DAPI (1:1,000 dilution) for 5 min at room temperature, and excess DAPI was removed with a final wash with PBS for 10 min. The fluorescently labelled BM tissue within bone fossae was placed cut-face down onto a 35-mm coverglass culture dish (MatTek Corporation), in (50–100 μl) PBS to prevent desiccation. Fluorescence images of as many as four colours were captured sequentially, using a 360-nm or 405-nm laser line and emission 385–470 nm for DAPI, a 488-nm laser line and emission between 488 and 522 nm for Alexa Fluor 488, a 555-nm laser line and emission between 555 and 585 nm for Cy3, and a 639-nm laser line and emission between 639 and 669 nm for Dylight 649. Series of *x*–*y*–*z* images of typically 640 × 640 μm^2^ *x*–*y* size were collected along the *z*-axis at 1- to 4-μm intervals to ∼100- to 150-μm depths throughout the BM tissue, using a × 10 objective. Whole-mount sterna imaged as indicated were analysed using the Imaris software (Bitplane; South Windsor) using built-in plugins to mask Collagen VI-bright vessels. Lineage^NEG^ cKit^HI^ progenitors were identified manually in each image and distances to vessels determined using automated algorithms.

For SLAM progenitor staining, femurs from necropsies were fixed with 4% paraformaldehyde in PBS at 4 °C overnight. Femoral bones were prepared and blocked as indicated above for sterna. The specimens were frozen in 2-methylbutane (Fisher Scientific), and cooled on dry ice, and 8-μm thick sections were cut in a cryostat. Tissues slides were blocked 20 min to prevent nonspecific binding and permeabilized with TNB 0.1% Triton at 4 °C. After three washes with PBS, we incubated at 4 °C for 2 days with anti-mouse CD48 biotin (110410 BioLegend 1:200 dilution), purified anti-mouse CD150 (115902 BioLegend 1:50 dilution), the rat mAb Lineage biotynilated cocktail and a rabbit anti-laminin antibody (L9393-2mL Sigma 1:200 dilution). After washing four times for 5 min with PBS plus Triton 0.05%, the samples were incubated with secondary antibodies (anti-rat IgG FITC (1/200 diluted), streptavidin 546 (1/100 diluted) and AlexaFluor 635 goat anti-rabbit (1/200 diluted)) at 4 °C overnight. Samples were then washed four times for 5 min. Nuclei were stained using Vectashield Mounting medium with DAPI (H1200 VectorLabs). All images were acquired with a Zeiss LSM 700 confocal system (Carl Zeiss MicroImaging) and analysed using the Fiji software with customized plugins.

### Chemokine and cytokine quantification

CXCL12 was measured from total BM extracellular fluid content obtained after flushing BM from femurs in 1 ml of PBS, using commercially available ELISA reagents (R&D Systems). Samples were diluted 1:2 in PBS. Levels of active TGFβ were quantified using Quantikine ELISA kit (R&D Systems) following the manufacturer's instructions. Samples were diluted 1:10 in PBS. For samples from *Glg1*^−/−^ mice, the values were corrected for the reduced number of cells and marrow size of the mutant mice.

### Western blotting

For western blot analyses, B and T lymphocytes from BM were stained with biotinylated anti-B220 (Clone: RA3.3A1/6.1) and anti-CD3e-APC (Clone: 145-2C11), respectively. Monocytes, neutrophils, macrophages, MP and LSK were stained as described above. Cells were sorted and lysed in Laemmli's sample buffer with 50 mM dithiothreitol, and boiled for 5 min. The equivalent of 10^5^ cells (for the myeloid compartment) or 1.5 × 10^5^ cells (for lymphocytes) was loaded onto each lane of a 7.5% SDS–PAGE gel and after electrophoresis the separated proteins were blotted onto a nitrocellulose membrane (Millipore). Membranes were blocked and incubated with an anti-ESL1-myc rabbit serum (a gift of Dr M.K. Wild; 1:1,000 diluted) and then washed thrice. The primary antibody was detected by using horseradish peroxidase (HRP)-conjugated anti-rabbit IgG (GE Healthcare; 1:5,000 diluted). Membranes were incubated with HRP substrate (Luminata Forte Western HRP substrate, Millipore) and luminescence was recorded on an ImageQuant LAS 4000 mini system (GE Healthcare). To obtain an internal load control, the membranes were then stripped and re-blotted with an anti-β-actin antibody (Sigma; 1:2,500 diluted).

### Progenitor assays in culture

BM were collected and 20,000 mononuclear cells were added to semisolid media containing 1.25% methylcellulose (Sigma-Aldrich), 30% FBS (StemCell Technologies), 1% deionized bovine serum albumin, 10^−4^ M 2-mercaptoethanol and conditioned medium (12.7% v/v) from the WEHI3 cell line (containing IL-3), HM-5 cell line (containing GM-CSF) and BHK/MKL cell line stably transfected to produce the secreted form of murine Kit Ligand/SCF. In some experiments, cells were plated in Methocult GF M3434 (StemCell Technologies) for identification of different types of precursors in culture (colony-forming unit granulocyte-macrophage (CFU-GM), colony-forming unit macrophage (CFU-M) and colony-forming unit granulocyte (CFU-G); burst-forming unit erythroid (BFU-E); colony-forming unit granulocyte, erythroid, macrophage and megakaryocyte (CFU-GEMM)). The TGFβR-I inhibitor LY-2157299 (kindly provided by Eli Lilly; Indiana) was added at a concentration of 500 nM. Control cultures were grown in the presence of dimethylsulphoxide (vehicle for the inhibitor). Cultures were plated in duplicates in 35 mm culture dishes (Nunc A/S) and incubated at 37 °C in 5% CO_2_. CFU-C were scored on day 6 or 7 using an inverted microscope.

### *In vitro* culture

Sorted LSK were cultivated overnight in StemSpan medium (Stem Cell Technologies) supplemented with 100 ng ml^−1^ rmTPO, 100 ng ml^−1^ rmFlt3 ligand, 50 ng ml^−1^ rmSCF, in the presence of anti-TGFβ or control antibody at 10 μg ml^−1^. Subsequently, 10 μM BrdU was added to the cultures for 4 h, and BrdU incorporation and cell surface phenotypes were analysed.

### RNA isolation and real-time quantitative PCR

BM LSKs were purified from femurs and tibiae. Staining was performance as described above. Total RNA was prepared with RNA Extraction RNeasy Plus Mini- or Micro-kit (Qiagen). RNA was reverse-transcribed with High-Capacity cDNA Reverse Transcription reagents kit (Applied Biosystems) according to the manufacturer's protocol. Real-time quantitative PCR (SYBR-green, Applied Biosystems) assays were performed with an Applied Biosystems 7900HT Fast Real-Time PCR System sequencer detector. Expression was normalized to *Hprt* (F-5′-CCTAAGATGAGCGCAAGTTGAA-3′, R-5′-CCACAGGACTAGAACACCTGCTAA-3′) and *36b4* (F-5′-ACTGGTCTAGGACCCGAGAAG-3′, R-5′-TCCCACCTTGTCTCCAGTCT-3′) expression. The primer sequences for *Glg-1* were: F-5′-CAAGATGACGGCCATCATTTT-3′, R-5′-TTCCCCAAGACGAATGCTGC-3′; for *p18*: F-5′-TTATGAAGCACACAGCCTGCAATGT-3′, R-5′-ACGGACAGCCAACCAACTAACGG-3′; for *p21*: F-5′-TGTCTTGCACTCTGGTGTCTGAGC-3′, R-5′-TCTTGCAGAAGACCAATCTGCG-3′; for *p57*: F-5′-GCGCAAACGTCTGAGATGAG-3′, R-5′-AGAGTTCTTCCATCGTCCGCT-3′; for *Tgfb1*: F-5′-CCGAAGCGGACTACTAT-3′; R-5′-GTAACGCCAGGAATTGT-3′.

### Statistical analyses

Data are shown as the mean+s.e.m. For comparisons between two groups the Student's *t*-test was applied. For data with more than two data sets, we used one-way analysis of variance with Turkey's multigroup test. Log-rank analysis was used for Kaplan–Meier survival curves. For the limiting dilution LT-reconstitution assays, we used Poisson's statistics as indicated. Analyses were performed with the GraphPad Prism software. *P* values<0.05 were deemed significant.

## Additional information

**How to cite this article:** Leiva, M. *et al*. Haematopoietic ESL-1 enables stem cell proliferation in the bone marrow by limiting TGFβ availability. *Nat. Commun.* 7:10222 doi: 10.1038/ncomms10222 (2016).

## Supplementary Material

Supplementary Information Supplementary Figures 1-10 and Supplementary Table 1
