# Supplementary material for: Haematopoietic ESL-1 enables stem cell proliferation in the bone marrow by limiting TGFβ availability
Source: Nat Commun. 2016 Jan 8;7:10222. doi: 10.1038/ncomms10222 (PMC4729861; doi:10.1038/ncomms10222)
Supplement: Supplementary Information — Supplementary Figures 1-10 and Supplementary Table 1 [file ncomms10222-s1.pdf]

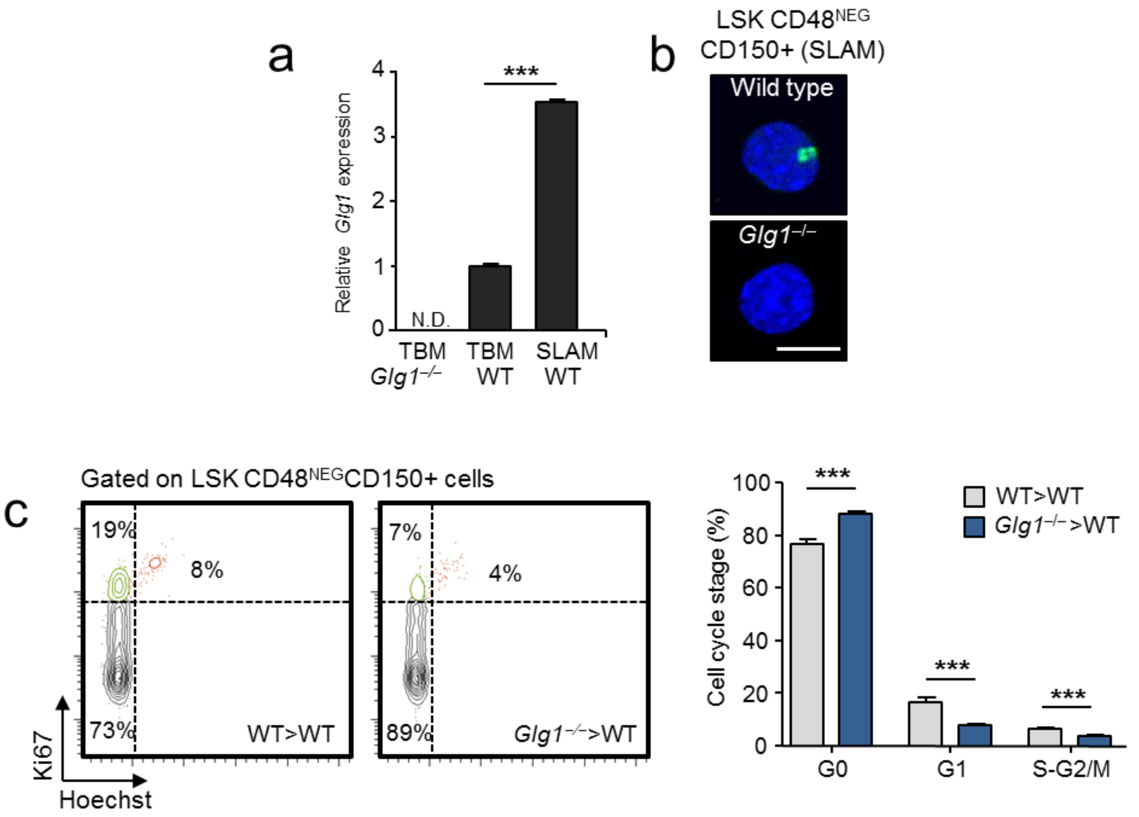

Supplementary Figure 1. Expression of ESL-1 on primitive progenitors. Primitive HSPC (LSK CD48^{NEG} CD150+; SLAM) were sort-purified from WT and *Glg1*-/- mice and analysed for expression of ESL-1. (a) Relative transcript levels of *Glg1* as determined by real-time qPCR of total bone marrow (TBM) or purified SLAM cells. n= 3; data shown as mean $\pm$ SEM. ND, not detectable. (b) Representative immunofluorescence images of purified SLAM cells showing the presence and distribution of ESL-1 (ESL-1, green; DAPI, blue). Scale bar, 5 μm. (c) Representative plots and quantification of cycling in WT or *Glg1*$^{-/-}$ SLAM progenitors as determined by staining with Ki67 and Hoechst 33342. Indicated are the percentages of cells in each quadrant for these samples. n=7 mice per group; ***p<0.001, as determined by the Student's test.

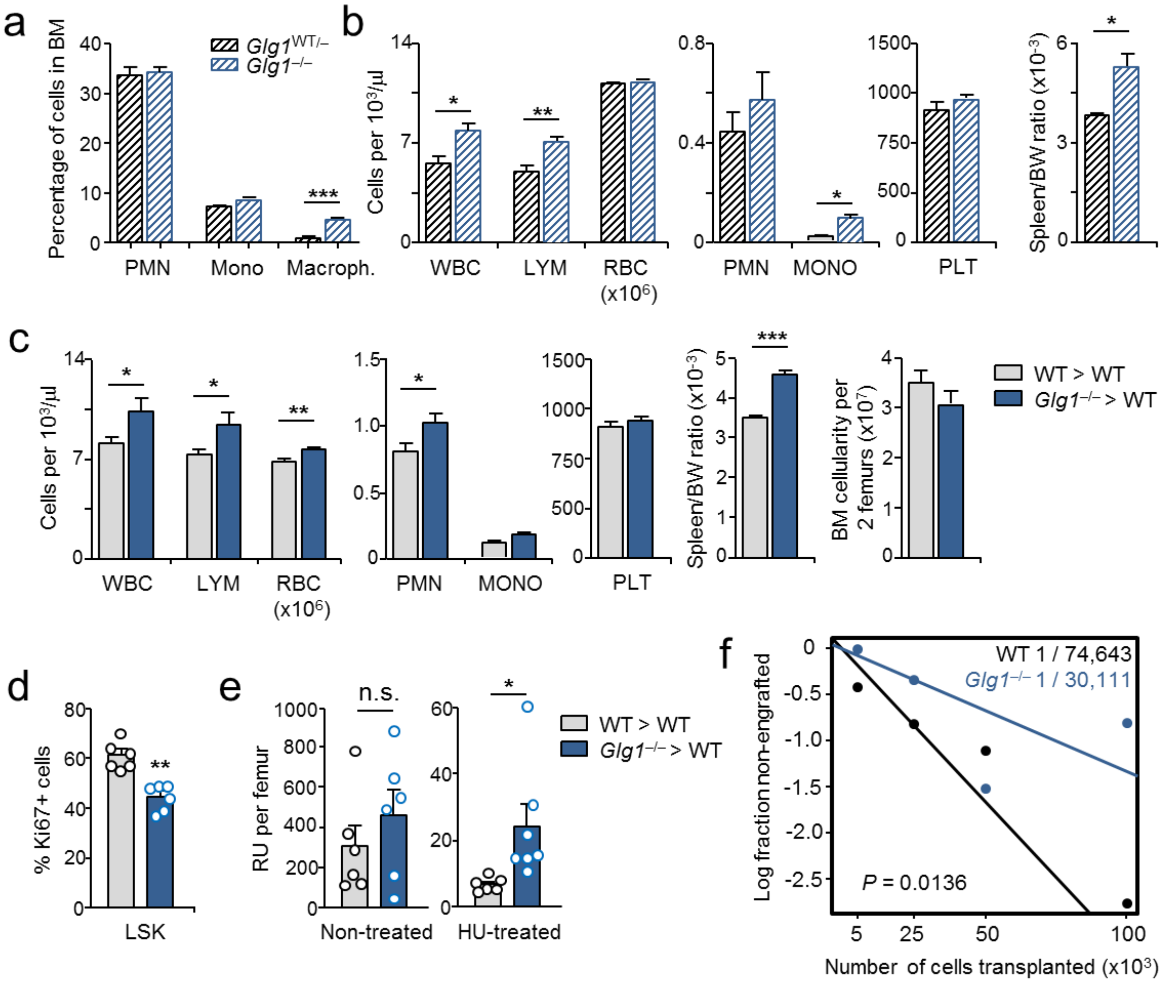

Supplementary Figure 2.Phenotypic and functional characterization of fully-deficient *Glg1*$^{-/-}$ mice or WT mice reconstituted with ESL-1-deficient bone marrow. (a) Percentage of myeloid cell subsets in the bone marrow (BM) of *Glg1*$^{-/-}$ and control littermates; n = 5. (b) Blood cell counts and spleen-to-body weight ratios of *Glg1*$^{-/-}$ and heterozygous littermates; n=4. (c) Blood counts, spleen-to-body weight ratio and BM cellularity in WT mice transplanted with WT or *Glg1*$^{-/-}$ donors; n=8-9. (d) Percentage of LSK cells that express the proliferation-related antigen Ki67 as determined by flow cytometry; n=6. (e) Number of reconstituting units per femur in mice transplanted with WT or *Glg1*$^{-/-}$ donors and treated or not with hydroxyurea; n=6-7. Note the different scales for each group. (f) Long-term competitive reconstitution assays using limiting dilutions of WT or *Glg1*$^{-/-}$ donor cells to estimate the frequency of functional HSC .n=9 per group and dose. Indicated are the frequencies and *P* value using Poisson statistics. *p<0.05; **p<0.02; ***p<0.001, as determined by the Student's test. Each circle represents one mouse. Data are shown as mean $\pm$ SEM.

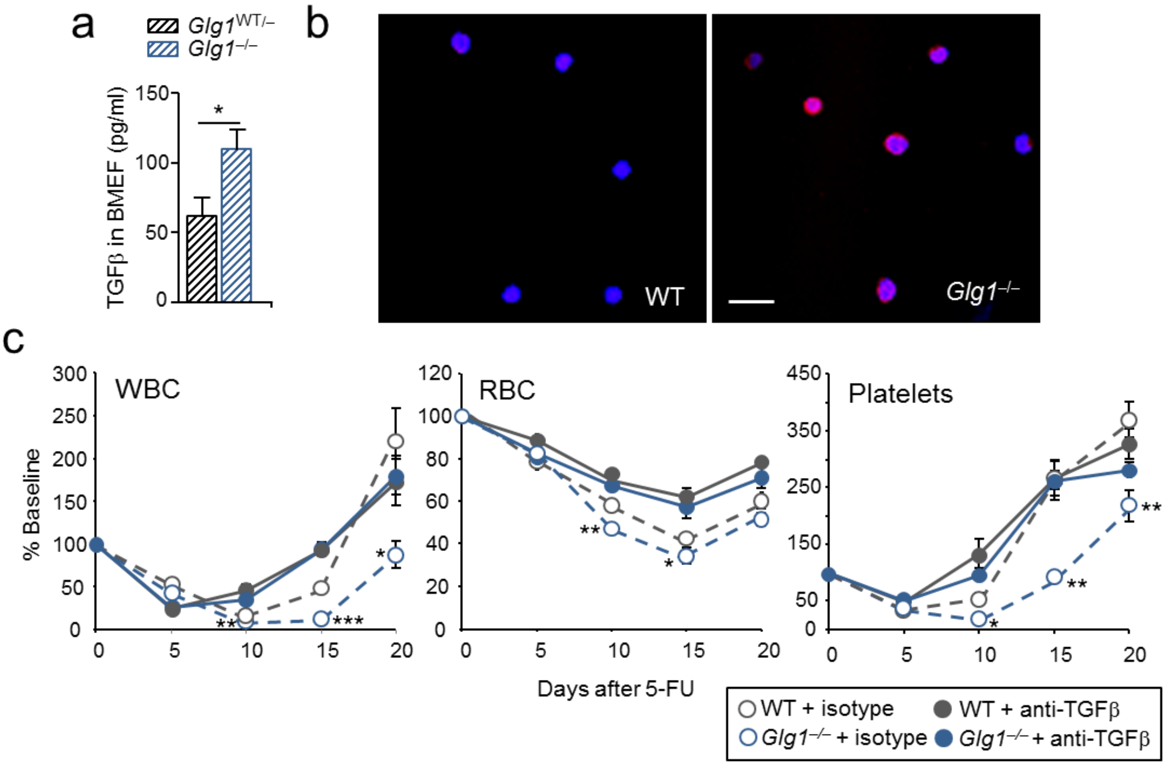

Supplementary Figure 3.

ESL-1 controls hematopoietic stem and progenitor cell (HSPC) proliferation through TGFβ. (a) ELISA measurement of active TGFβ in the bone marrow (BM) of *Glg1*$^{-/-}$ mice and littermate heterozygous controls; n=4. **(b)** Cytospun samples of sorted LSK stained with anti-phospho-Smad2/3. Scale bar, 10 μm. **(c)** Kinetics of recovery for total leukocytes (WBC), red blood cells (RBC) and platelets after 5-FU treatment in mice transplanted with WT or *Glg1*$^{-/-}$ donors, and treated with isotype or anti-TGFβ antibodies; n=6 mice per group. Data are shown as mean ± SEM. *p<0.05; **p<0.01; ***, p<0.001, as determined by the Student's test. For panels in **(c)**, comparisons are *Glg1*$^{-/-}$ isotype vs. WT isotype, and *Glg1*$^{-/-}$ + anti-TGFβ vs. WT + anti-TGFβ. No differences were found between the anti-TGFβ groups at any time point.

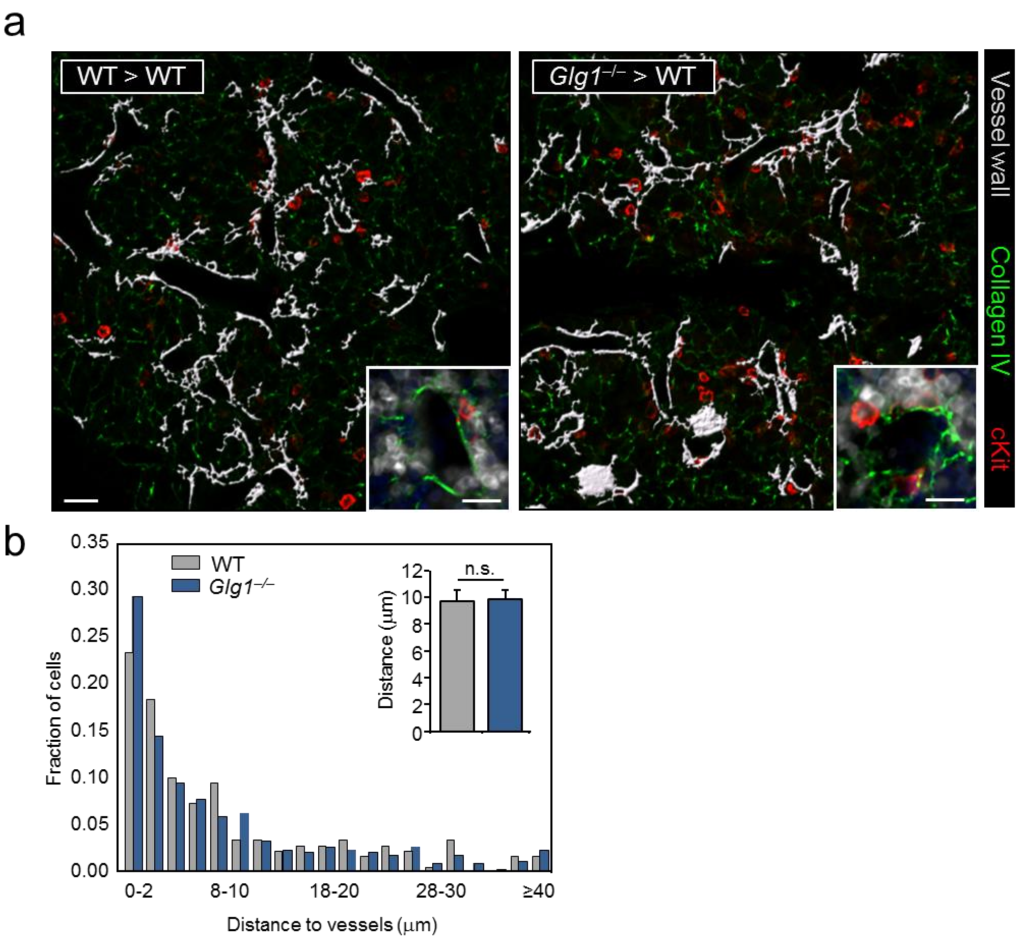

Supplementary Figure 4.
ESL-1 does not alter the localization of hematopoietic progenitors. (a) Representative whole-mount images and distribution of Lin^{NEG}cKit+ cells in the sternal marrow relative to collagen^{HI} (white contours) vessels where Lineage staining has been removed for clarity; scale bar, 50μm. Insets show details of Lin^{NEG}cKit+ cells relative to vessel structures, with Lineage staining in white; scale bar, 10μm; n=4-7 mice,180-337 progenitor cells per group. **(b)** Distribution of the distances of Lin^{NEG}cKit+ cells to vessels in WT or *Glg1*$^{-/-}$ bone marrow (BM). n.s., not significant, as determined by Student's t-test. Bars in inset show mean $\pm$ SEM fraction of cells at the indicated distances to vessels.

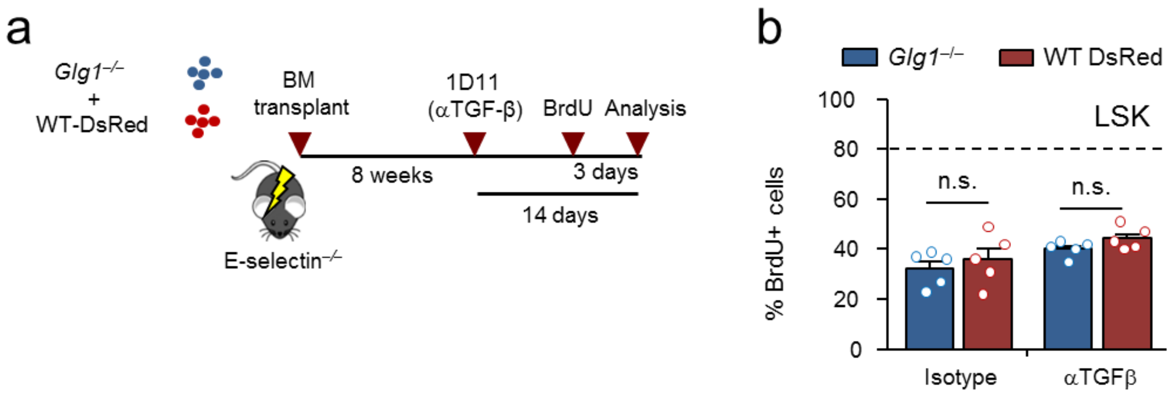

Supplementary Figure 5.

ESL-1 and E-selectin create a proliferative niche through independent mechanisms.

Lethally irradiated E-selectin-deficient mice were transplanted with a mix of *Glg1*$^{-/-}$ and WT-DsRed bone marrow (BM). 8-weeks after transplant mice were treated with an anti-TGFβ (clone 1D11) or an isotype control antibody. BrdU incorporation was assessed after 2 weeks of treatment. Right, percentages of BrdU+ LSK cells. For reference, the dashed line indicates the percentage of BrdU LSK cells in untreated WT mice (Figure 2C); n=5. n.s., not significant as determined by impaired Student’s test. Each circle represents a mouse. Data shown as mean + SEM.

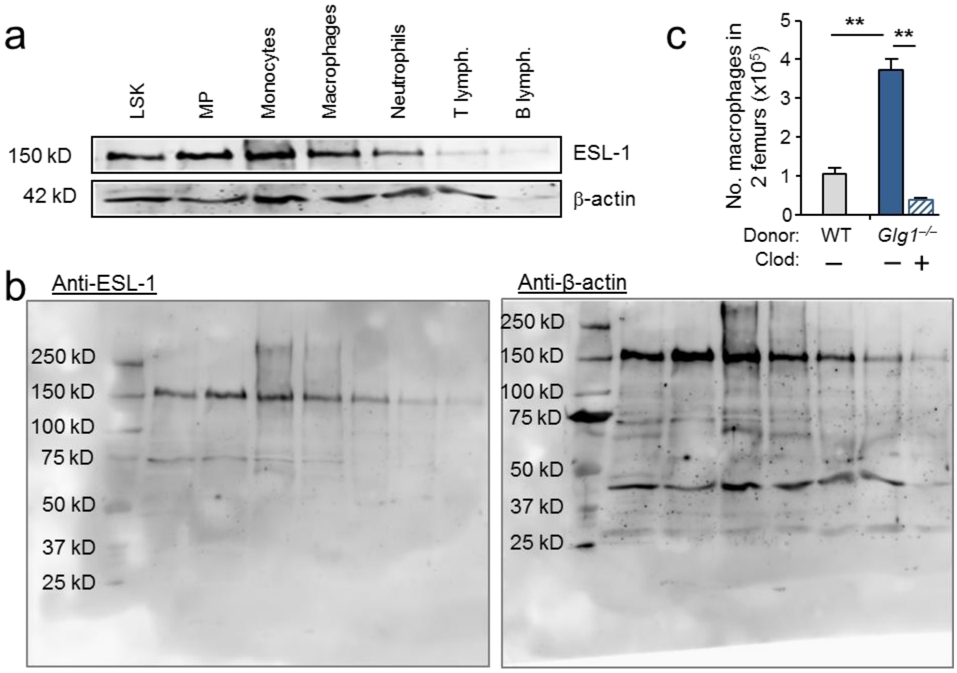

Supplementary Figure 6.

ESL-1 levels in hematopoietic subsets and depletion of macrophages. (a) Representative immunoblots of ESL-1 and β–actin (load control) in the indicated FACS-purified BM populations (1.5x10^5 cells/lane). Data representative of 2 independent experiments. (b) Images of the original western blot membranes. (c) Absolute numbers of BM macrophages per 2 femurs in mice transplanted with WT and *Glg1*$^{-/-}$ BM and treated with clodronate (Clod) or vehicle; n= 4 mice per group. ;**p<0.01 as determined by ANOVA with Tukey's multigroup test. Data are shown as mean $\pm$ SEM.

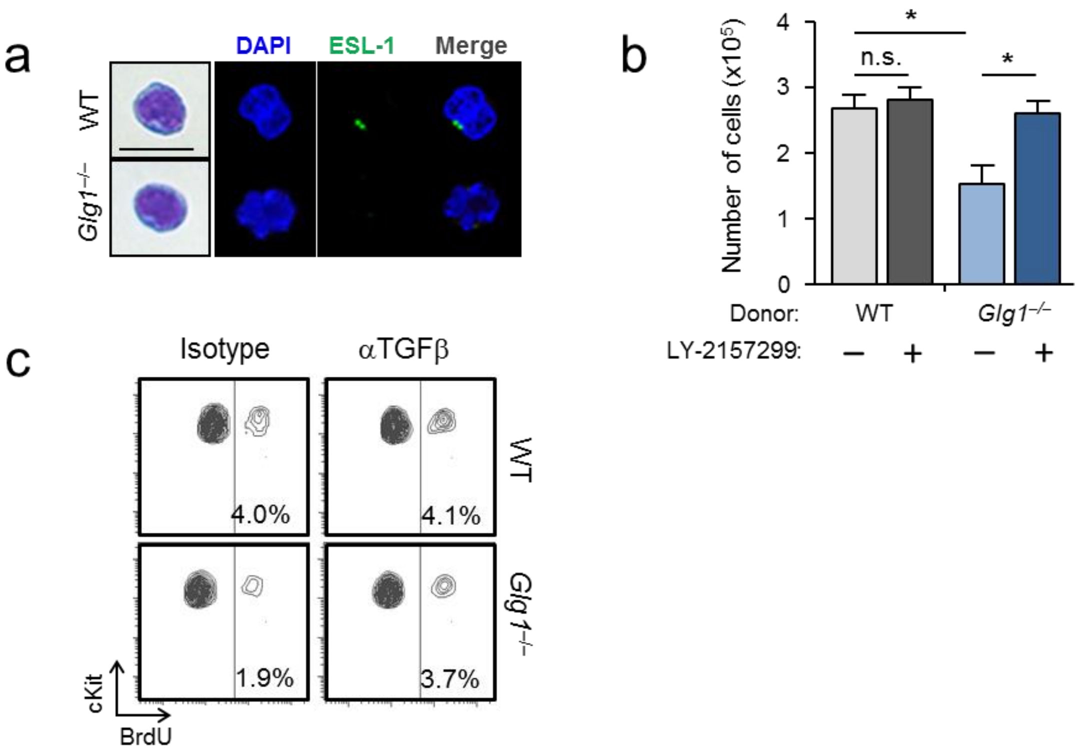

Supplementary Figure 7.

ESL-1-deficient hematopoietic precursors are a relevant source of TGFβ. (a) Giemsa and immunofluorescence staining of ESL-1 in WT and *Glg1*$^{-/-}$ LSK cells; scale bar 5 μm. (b) Bar graphs show cell counts of CFU-C cultures and exposed or not to LY-2157299; data from 3 independent experiments. (c) Contour plots illustrating the incorporation of BrdU in FACS-purified WT and *Glg1*$^{-/-}$ LSK cells cultured overnight in presence or absence of a blocking anti-TGFβ antibody.

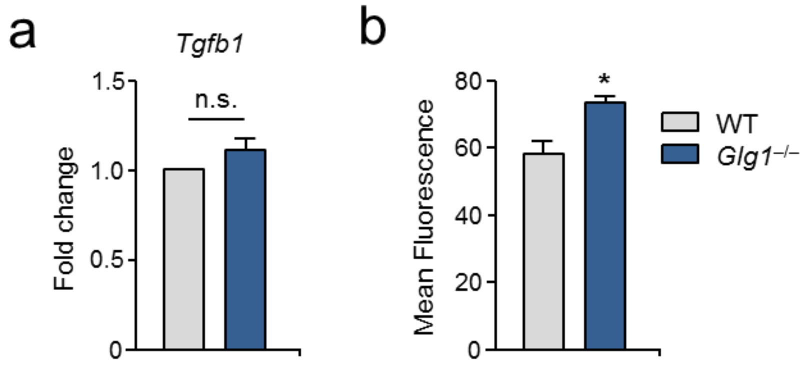

Supplementary Figure 8.

Transcript and protein levels of TGFβ in hematopoietic precursors. (a) Relative expression of the *Tgfb1* gene in WT and *Glg1*$^{-/-}$ LSK cells. (b) Fluorescence levels of latent TGFβ in WT and *Glg1*$^{-/-}$ LSK cells. Fluorescence background in the presence of an isotype control was subtracted from the MFI of each sample; n=3. n.s., not significant; *p<0.05 as determined by Student's t-test. Data are shown as mean $\pm$ SEM.

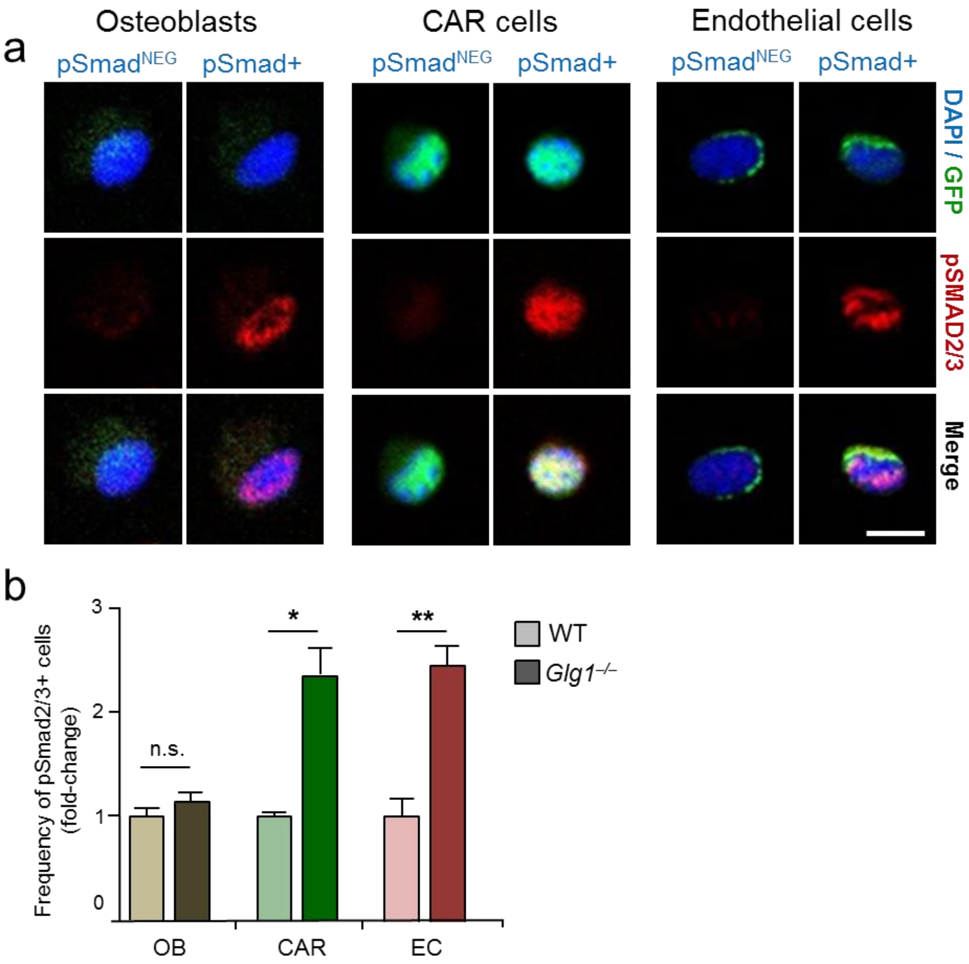

Supplementary Figure 9.

Activation of the TGFβ signalling pathway in endothelial and CXCL12-abundant reticular (CAR) cells exposed to *Glg1*$^{-/-}$ hematopoietic cells. (a) Representative images of sorted osteoblasts (OB), endothelial cells (EC) and CAR cells from *Cxcl12*^{GFP} mice transplanted with WT or *Glg1*$^{-/-}$ BM, and stained with an anti-pSMAD2/3 antibody. Note the varying levels of GFP among the different cell types. Scale bar, 10 µm. (b) Relative frequency of sorted cells of the different subsets positive for pSMAD2/3. Data is from 42-51 (CAR), 125-155 (OB) and 180-213 (EC) cells per group, from 2 independent experiments. n.s., not significant; *p<0.05, **, p<0.01 as determined by Student's t-test. Data are shown as mean ± SEM.

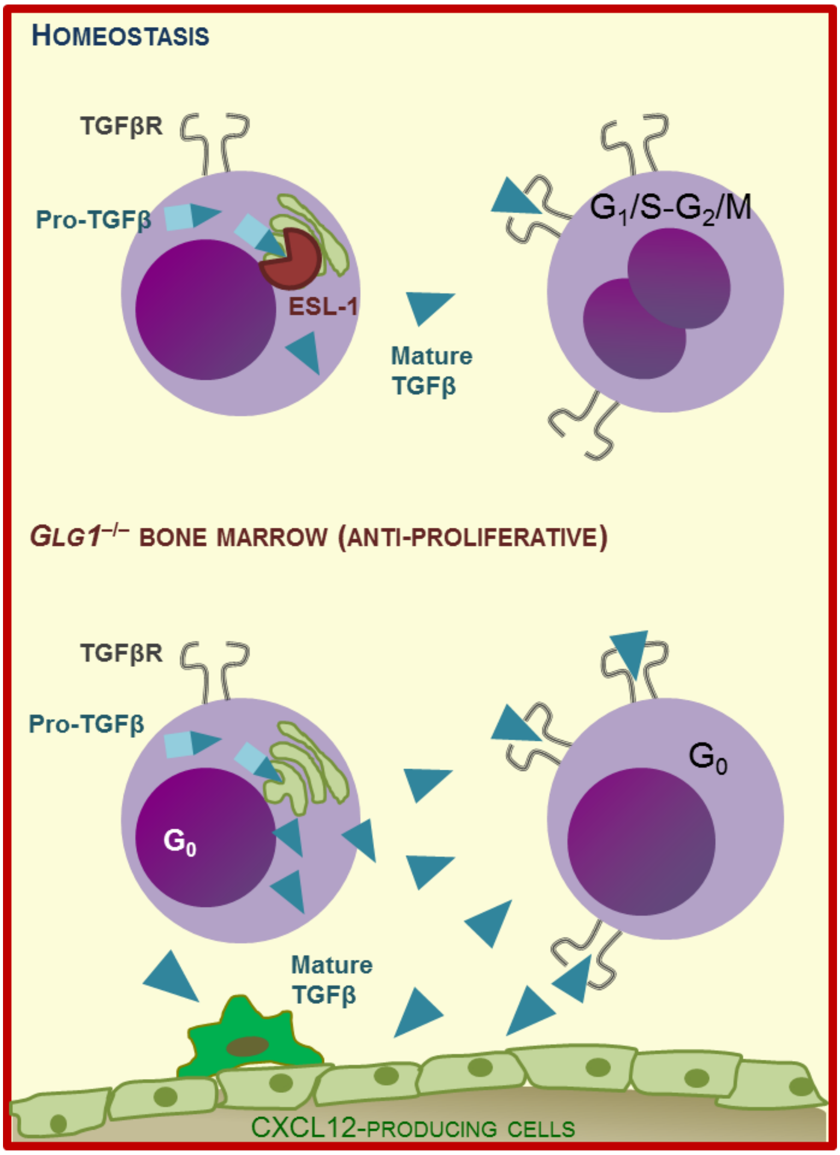

Supplementary Figure 10.
ESL-1 enables hematopoietic stem and progenitor cell (HSPC) proliferation in the bone marrow (BM) by limiting TGFβ availability. Under homeostatic conditions, ESL-1 limits TGFβ release from BM cells, including primitive hematopoietic progenitors, thus favoring proliferation and increased susceptibility to genotoxic injury. Consequently, absence of ESL-1 results in increased levels of the cytokine in the local niche environment, leading to enhanced cell cycle arrest in neighboring HSPC and reduced number of the stromal elements that produce CXCL12 (green cells).

Supplementary Table 1. A list of antibodies used in this study.

Antibody	Dilution (per 10^6 cells)	Clone	Source
Gr1-FITC	0.2 µg	RB6-8C5	eBioscience
CD115-PE	0.05 µg	AFS98	eBioscience
F4/80-APC	0.1 µg	A3.1	Serotec
Lineage-biotin cocktail	5 µl	Several clones	BD
Sca-1-FITC	0.1 µg	D7	eBioscience
Sca-1-APC	0.05 µg	D7	eBioscience
c-Kit-PE-Cy7	0.05 µg	2B8	eBioscience
CD135-PE	0.05 µg	A2F10	eBioscience
CD90.2-APC	0.05 µg	53-2.1	eBioscience
CD48-biotin	0.1 µg	HM48-1	Biolegend
CD150-PE	0.05 µg	TC15-12F12.2	Biolegend
CD45.2-FITC	0.5 µg	104	Tombo
CD45.1-PE-Cy7	0.5 µg	A20	Southern Biotech
CD11b-PE	0.2 µg	M1/70	eBioscience
CD3ε-APC	0.2 µg	145-2C11	Biolegend
B220-biotin	0.2 µg	RA3.3A1/6.1	BD
BrdU-APC	0.2 µg	N/A	BD
Ki67-APC	0.2 µg	SolA15	eBioscience
c-Kit-APC	0.05 µg	2B8	eBioscience
CD45-biotin	0.5 µg	30-F11	eBioscience
CD31-APC	0.2 µg	390	eBioscience
TER119-biotin	0.2 µg	TER119	BioXcell
Isotype-APC	0.1 µg	MOPC-21	Biolegend
LAP-APC	0.1 µg	TW7-16B4	Biolegend
Streptavidin-APC-Cy7	0.4 µg	-	eBioscience
Streptavidin-DyLight 405	0.3 µg	-	Jackson

N/A, not available
